# Small RNAs with 5′-Polyphosphate Termini Associate with a Piwi-Related Protein and Regulate Gene Expression in the Single-Celled Eukaryote *Entamoeba histolytica*

**DOI:** 10.1371/journal.ppat.1000219

**Published:** 2008-11-28

**Authors:** Hanbang Zhang, Gretchen M. Ehrenkaufer, Justine M. Pompey, Jason A. Hackney, Upinder Singh

**Affiliations:** 1 Division of Infectious Diseases, Department of Internal Medicine, Stanford University School of Medicine, Stanford, California, United States of America; 2 Department of Microbiology and Immunology, Stanford University School of Medicine, Stanford, California, United States of America; University of California Los Angeles, United States of America

## Abstract

Small interfering RNAs regulate gene expression in diverse biological processes, including heterochromatin formation and DNA elimination, developmental regulation, and cell differentiation. In the single-celled eukaryote *Entamoeba histolytica*, we have identified a population of small RNAs of 27 nt size that (i) have 5′-polyphosphate termini, (ii) map antisense to genes, and (iii) associate with an *E. histolytica* Piwi-related protein. Whole genome microarray expression analysis revealed that essentially all genes to which antisense small RNAs map were not expressed under trophozoite conditions, the parasite stage from which the small RNAs were cloned. However, a number of these genes were expressed in other *E. histolytica* strains with an inverse correlation between small RNA and gene expression level, suggesting that these small RNAs mediate silencing of the cognate gene. Overall, our results demonstrate that *E. histolytica* has an abundant 27 nt small RNA population, with features similar to secondary siRNAs from *C. elegans*, and which appear to regulate gene expression. These data indicate that a silencing pathway mediated by 5′-polyphosphate siRNAs extends to single-celled eukaryotic organisms.

## Author Summary

Regulation of gene expression can occur via multiple conserved pathways. One such mechanism is mediated by RNA molecules of about 21–24 nucleotides (called small RNAs), which can affect rates of RNA degradation or protein production. These small RNA molecules regulate diverse biological processes in a broad range of systems. The vast majority of the published literature about these molecules is from multi-cellular organisms. We have made a number of novel observations with respect to small RNA size, structure, and function in *Entamoeba histolytica*, a single-celled parasite and an important human pathogen. Our work has identified that *E. histolytica* has an abundant population of 27 nucleotide small RNAs, which have an unusual structure, indicating that they are generated by a relatively atypical mechanism. A substantial portion of these small RNAs are antisense to target genes and appear to silence them. These data establish a new paradigm for how gene expression is regulated in this organism. Furthermore, the identification of small RNAs with these structural characteristics dramatically broadens the evolutionary spectrum in which this phenomenon has been identified and indicates significant diversity and complexity of small RNAs and their functions in single-celled eukaryotes.

## Introduction

Small RNAs mediate post-transcriptional gene silencing in a multitude of organisms and in diverse biological processes [1],[2],[3],[4],[5]. Two proteins central to the small RNA mediated gene silencing pathways are Dicer, an RNaseIII enzyme, which generates small RNAs and Argonaute, which associates with the small RNAs and target genes to mediate gene silencing. Multiple classes of small RNAs have recently been described including small interfering RNAs (siRNAs), microRNAs (miRNAs), trans-acting siRNAs (tasiRNAs), tiny noncoding RNAs (tncRNAs), small scan RNA (scRNA), repeat-associated small interfering RNA (rasiRNA), piwi-interacting RNA (piRNA), and secondary siRNAs [6],[7],[8],[9]. Some organisms have multiple populations of small RNAs associated with different mechanisms of gene regulation. Notably siRNAs, miRNAs, tasiRNAs, tncRNA, and scnRNA are all products of Dicer cleavage. In contrast, rasiRNA, piRNA, and secondary siRNA appear to be formed independent of Dicer processing [6],[7],[8],[10].

Primary siRNAs are produced from long double stranded RNAs and can be endogenously derived from repetitive genomic regions, transposon elements, or regions with active antisense transcripts. Primary siRNAs are generated by Dicer processing, which generates a 5′-monophosphate (5′-monoP) and 3′-hydroxyl (3′-OH) structure. Primary siRNAs are subsequently loaded into Argonaute to mediate gene silencing but can also serve as the “trigger” to initiate RNA-dependent RNA polymerase (RdRP) generation of secondary siRNAs. In plants, secondary siRNAs, although generated by RdRP, are eventually processed by Dicer and thus the majority have the classic 5′-monoP termini [9]. In *C. elegans*, secondary siRNAs have a 5′-polyphosphate (5′-polyP) structure, a feature not identified in any other siRNAs to date. *C. elegans* secondary siRNAs largely map antisense to genes, are biased towards the 5′ side of primary trigger RNAs, and amplify gene silencing by their association with CSR-1, an Argonaute protein [7],[8],[10],[11],[12]. Because of their 5′-polyP structure, *C. elegans* secondary siRNAs are most efficiently cloned in a 5′-phosphate independent manner [7],[8].

*Entamoeba histolytica*, a single celled eukaryote, is an important human pathogen and a leading parasitic cause of death worldwide [13]. The parasite has two stages in its life cycle: an invasive trophozoite form, which causes disease and a dormant cyst form, which transmits disease [14]. The genome of *E. histolytica* is predicted to encode a number of genes conserved in the RNAi pathway including three genes with Piwi and PAZ domains (EHI_186850, EHI_125650, and EHI_177170), and two genes with RdRP domains (EHI_139420 and EHI_179800) [15]. However, no obvious homologue of Dicer was identified, although RNaseIII activity was detected in *E. histolytica* trophozoites and a protein with an RNaseIII domain (EHI_068740) has been identified in the genome sequence [15],[16]. Recently it has been shown that dsRNA, siRNA, and short-hairpin RNAs are effective in achieving gene silencing in *E. histolytica* suggesting that the machinery for small RNA mediated silencing is functional in this parasite [17],[18],[19]. Additionally, putative microRNAs were identified in *E. histolytica* using a bioinformatics-based approach, however, none of those predictions were confirmed using high resolution Northern blot analysis [20]. Thus, although there are hints that a functional RNAi pathway exits in *E. histolytica*, endogenous small RNAs have not previously been identified in this eukaryotic pathogen.

In order to identify endogenous small RNAs in *E. histolytica*, we used a 5′-phosphate independent cloning method to clone small RNAs from *E. histolytica* trophozoites. Our analysis identified an abundant population of small RNAs (∼27nt) with features highly reminiscent of secondary siRNAs from *C. elegans*. *E. histolytica* 27 nt small RNAs have 5′-polyphosphate termini, largely map antisense to genes with bias towards the 5′ ends of genes, are associated with a Piwi-related protein, and appear to regulate strain-specific gene expression. Thus, *Entamoeba histolytica*, an organism typically considered a simple eukaryote, appears to use complex regulatory mechanisms to mediate gene silencing. Even though there are some important differences between these small RNAs in *E. histolytica* and *C. elegans* (including the size of the 5′-polyP small RNAs), our data indicate that the secondary siRNA mechanism of gene silencing extends deep into the evolutionary spectrum.

## Results

### Identification of 27 nt small RNA population with 5′-polyphosphate termini in *Entamoeba histolytica* trophozoites

**Figure 1:**
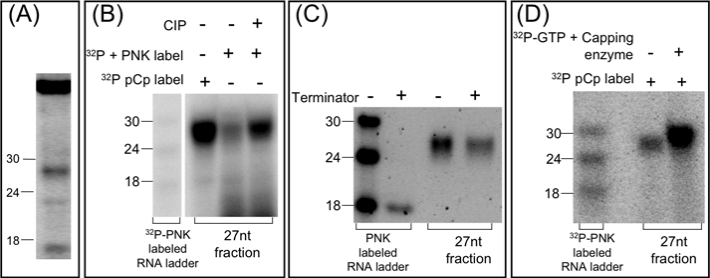
27 nt small RNAs in *Entamoeba histolytica* trophozoites have 5′-polyphosphate termini. (A) Three endogenous small RNA populations (∼27 nt, ∼22 nt, and ∼16 nt) can be detected in *Entamoeba histolytica* trophozoites by SYBR gold staining. The 27 nt population is the most abundant. (B) The 27 nt small RNA fraction can be labeled at the 3′-termini with α-^32^P pCp but does not label well with PNK (at the 5′-termini) unless the sample is first dephosphorylated by treatment with CIP. (C) A significant portion of the 27 nt small RNA fraction is resistant to cleavage by Terminator enzyme. The control (RNA ladder with a 5′-monophosphate) is degraded by Terminator enzyme. (D) The 27 nt small RNA fraction labels and increases in size when treated with ^32^P-GTP+Capping enzyme, indicating that the 27 nt small RNAs have 5′ di- or tri-phosphate termini.

In order to visualize small RNAs in *E. histolytica* trophozoites, we fractionated total RNA with a YM100 column and visualized the samples on 12% denaturing polyacrylamide gel stained with SYBR gold. Three distinct populations of small RNAs were visualized: ∼27 nt, ∼22 nt, and ∼16 nt with the 27 nt population the most abundant (Figure 1A). A similar pattern was seen in trophozoites of the non-invasive human parasite *Entamoeba dispar* (ED), and the reptilian parasite *Entamoeba invadens* (EI) (Figure S1). Thus, it appears that the overall pattern of three distinct small RNA populations is conserved in *Entamoeba* species.

Since the 27 nt small RNA population was the most abundant we subsequently focused our efforts on this population. We first attempted to define the features of the 5′ and 3′ termini of this small RNA population. We identified that the *E. histolytica* 27 nt fraction is likely to have a 3′-OH, as it can be efficiently labeled using RNA ligase and cytidine 3′,5′-bisphosphate [5′-^32^P] (abbreviated ^32^pCp hereafter) (Figure 1B). By contrast, the 5′ end is not likely to be a 5′-monoP, as it could not be efficiently labeled using polynucleotide kinase (PNK), unless first treated with calf intestinal phosphatase (CIP) (Figure 1B). To further confirm that the 5′ termini was not a 5′-monoP, we took advantage of the specificity of Terminator exonuclease for 5′-monoP RNA species: 5′-monoP RNAs are efficiently degraded by treatment with Terminator (a 5′-3′ exonuclease), whereas RNAs with a 5′-cap, 5′-polyP, or 5′-OH will be resistant. A substantial portion of the 27 nt small RNA population was resistant to treatment with Terminator enzyme, whereas the control (PNK labeled RNA ladder with 5′-monoP termini) was largely degraded by Terminator treatment (Figure 1C). Finally, treatment of the 27 nt fraction with a capping enzyme (which only caps RNAs with 5′ termini containing di- or tri-phosphate structures) increases the signal and the size of the 27 nt fraction (Figure 1D). Collectively, these data indicate that a substantial portion of the *E. histolytica* trophozoite 27 nt small RNAs have 5′-polyP termini. Some of the 27 nt population may be 5′-monoP species, as some RNA was labeled with PNK and there was a slight reduction in RNA abundance after treatment with Terminator enzyme.

### 5′-phosphate independent and dependent cloning of 27 nt small RNAs from *E. histolytica* trophozoites

**Figure 2:**
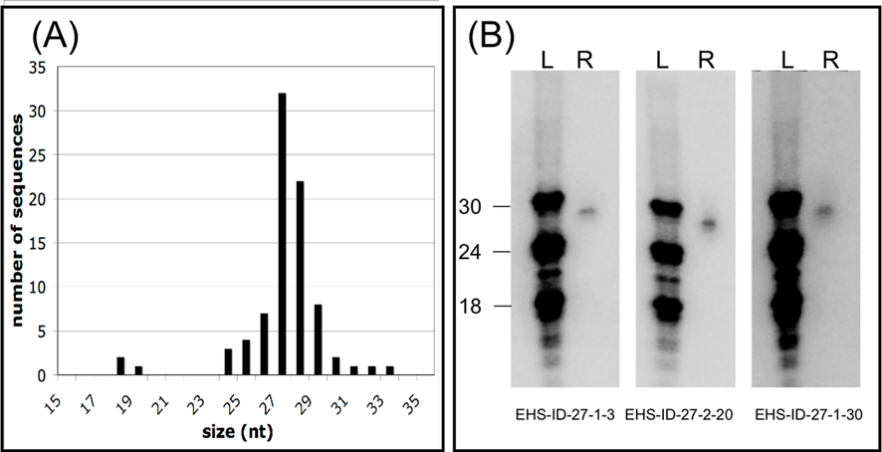
The 27 nt small RNAs can be cloned in a 5′P-independent manner. Using a 5′-phosphate independent cloning method the 27 nt RNA fraction was purified and small RNAs cloned and sequenced. (A) The size distribution of the cloned small RNAs. The number of cloned small RNAs is indicated on the y-axis and the size of the cloned RNAs is listed on the x-axis. (B) Cloned small RNAs can be detected at the appropriate size by Northern blot analysis. 10 µg of total RNA from *E. histolytica* trophozoites was probed with end-labeled ^32^P oligonucleotides corresponding to the cloned small RNAs. L = ladder; R = RNA.

The standard method for small RNA cloning depends on the presence of a 5′-monoP on the small RNA of interest [10]. However, this 5′-phosphate dependent method of cloning will not efficiently capture small RNAs with a 5′-polyP structure. In order to clone 27 nt small RNAs with 5′-polyP termini from *E. histolytica* trophozoites, we utilized a 5′-phosphate independent method of cloning and performed limited sequencing [8]. A total of 289 small RNA sequences were obtained, 243 of which were unique, and 196 of which mapped to the *E. histolytica* genome sequence (Table S1 and Figure S2). Overall, 27% of small RNAs mapped to ribosomal RNAs, 27% mapped to tRNAs, 20% mapped antisense to ORFs, 13% mapped to intergenic regions, 5% mapped to multiple genomic loci, 3% mapped sense to ORFs, 5% mapped to repetitive regions, and 0.5% mapped to retrotransposon elements. The size distribution of the cloned small RNAs peaked at 27–28 nt, matching the size of the population from which they were cloned (Figure 2A).

We also cloned small RNAs from a 15–30 nt size selected fraction from *E. histolytica* trophozoites using a 5′-phosphate dependent method; 802 small RNA sequences were obtained, 544 of which were unique, and 342 of which mapped to the *E. histolytica* genome sequence (Table S1 and Figure S2). The majority of the cloned RNAs were smaller in size with a peak size at ∼16 nt. However, high resolution Northern blot analysis demonstrated that small RNAs cloned in a 5′-phosphate dependent manner all mapped at ∼27–32 nt, regardless of the size at which they were cloned, indicating that we had cloned partial degradation products of the 27 nt small RNAs (Figure S3). In contrast, small RNAs cloned by the 5′-phosphate independent manner were detected by Northern blot analysis at sizes matching the sizes of the cloned products (Figure 2B). This confirms that the full length *E. histolytica* 27 nt small RNAs with 5′-polyP termini are not efficiently cloned using a 5′-phosphate dependent approach, while full length small RNAs can be cloned using a 5′-phosphate independent method. To confirm that we had not cloned degradation fragments of larger transcripts, we used Northern blot analysis on total RNA using probes for a number of cloned small RNAs and did not detect any signal >200 nt (data not shown). Thus, the majority of small RNAs that map antisense to genes do not appear to be degradation products of larger antisense transcripts.

**Figure 3:**
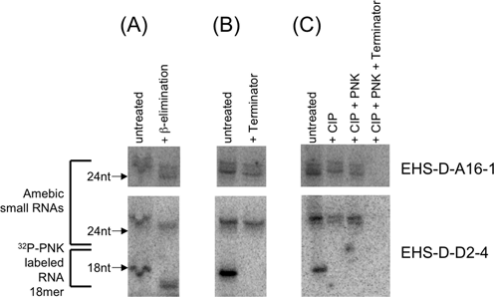
*E. histolytica* cloned 27 nt small RNAs have 5′-polyphosphate and 3′-OH termini. Biochemical analysis of the 5′ and 3′ termini of amebic cloned small RNAs. Probes detecting two 27 nt amebic RNAs (EHS-D-A16-1 and EHS-D-D2-4) are shown. An 18 nt 5′-monophosphate and 3′-OH radiolabeled probe is included as a control. (A) β-elimination reaction of *E. histolytica* small RNAs results in smaller species indicating that the 3′ termini of the small RNAs have an unmodified 3′-OH. (B) *E. histolytica* 27 nt small RNAs are resistant to treatment with Terminator enzyme indicating that the 27 nt RNAs do not have a 5′-monophosphate structure. (C) Small RNAs treated with CIP+PNK are susceptible to degradation with Terminator enzyme suggesting most likely a 5′-polyphosphate structure.

In order to confirm the 5′ and 3′ structure of the cloned 27 nt small RNAs, we tested individual small RNAs that had been identified in our library using biochemical approaches. The *E. histolytica* 27 nt cloned small RNAs are likely to have a free 3′-OH, as they are susceptible to loss of a single base and generation of a smaller RNA species following a β-elimination reaction, as indicated by faster migration following denaturing PAGE (Figure 3A). A ^32^P-PNK labeled synthetic RNA 18mer (with a 3′-OH terminus) also loses a base after a β-elimination reaction, as indicated by faster migration. In agreement with previous analyses of the whole 27 nt population, individual 27 nt small RNAs are initially resistant to Terminator treatment (Figure 3B), but become sensitive to Terminator exonuclease activity after CIP treatment followed by addition of a 5′-monoP by PNK (Figure 3C). The control synthetic 18mer RNA species, with a 5′-monoP is degraded by Terminator treatment (Figure 3B). We tested three different 27 nt small RNAs and found the same end structures for all three (some data not shown). These results confirmed that small RNAs cloned from the 27 nt population have a 5′-polyP and 3′-OH structure. Thus, the 27 nt small RNAs in *E. histolytica* are likely not Dicer products (based on 5′-polyP termini). Instead, the structure of these RNAs matches that of secondary siRNAs in *C. elegans*, the only siRNA species identified to date with 5′-polyP termini, and which are known to be products of RdRP processing [7],[8]. One major difference between the *E. histolytica* and *C. elegans* 5′-polyP small RNAs is the size of the populations: the *E. histolytica* population is ∼27 nt, whereas the *C. elegans* 5′-polyP small RNAs are ∼22 nt. The molecular mechanism that determines the sizes of these small RNAs in either system is not currently known.

### *E. histolytica* 27 nt 5′-polyphosphate small RNAs are in a complex with a Piwi-related protein

**Figure 4:**
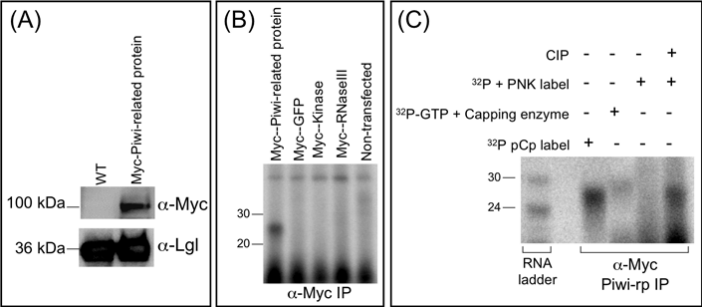
27 nt polyphosphate small RNAs associate with *E. histolytica* Piwi-related protein. (A) Western blot analysis of Myc-EhPiwi-rp. Using an anti-Myc antibody, signal is detected at ∼100 kD in the Myc-EhPiwi-rp transfectants. No signal is detected in the extract made from untransfected cells. An antibody to the light subunit of the *E. histolytica* surface lectin (Lgl) was used as a loading control. (B) Immunoprecipitation was performed with α-Myc antibody on parasites stably transfected with Myc-EhPiwi-rp, Myc-GFP, Myc-EhKinase, Myc-EhRNaseIII, and non-transfected parasites. RNA was extracted, labeled with α-P^32^ pCp and resolved on a denaturing gel. A ∼27 nt small RNA population was specifically identified as immunoprecipitating in the Myc-EhPiwi-rp cell lines. (C) Small RNAs that IP with EhPiwi-rp can be labeled at the 3′ end (α-P^32^ pCp), but not at the 5′ end (PNK), unless first treated with CIP. A capping enzyme labels the immunoprecipitated samples indicating that the EhPiwi-rp associated 27 nt small RNAs have 5′ di- or triphosphate termini.

In order to determine if the 27 nt small RNAs are involved in a silencing complex, we tested to see if they associate with *E. histolytica* Piwi-related protein (EhPiwi-rp) (EHI_125650). Of the three genes in the *E. histolytica* genome that are predicted to contain PIWI domains, this is the only one that is highly expressed in trophozoites [21]. A construct with N-terminal Myc tagged EhPiwi-rp was generated and transgenic parasites selected. Western blot analysis demonstrated that anti-Myc antibody detected a protein of the appropriate molecular mass specifically in the Myc-EhPiwi-rp transgenic strains (Figure 4A). Immunoprecipitation (IP) experiments with α-Myc antibody were performed and small RNAs of ∼27 nt were specifically observed in the α-Myc IP of the Myc-EhPiwi-rp parasite cell line (Figure 4B). The 27 nt small RNA population was significantly enriched in the α-Myc EhPiwi-rp immunoprecipitated sample compared to the starting sample (data not shown). No small RNAs immunoprecipitated with a number of controls including untransfected parasites or transgenic *E. histolytica* strains expressing one of a number of Myc-tagged proteins (Green Fluorescent Protein, EhKinase, or EhRNaseIII) (Figure 4B). These data indicate that 27 nt small RNAs are in a complex specifically with EhPiwi-rp.

In order to determine the 5′ and 3′ structure of the small RNAs that immunoprecipitated with Myc-EhPiwi-rp, we performed IP with α-Myc and used the biochemical approaches outlined earlier. In agreement with our analysis of the total 27 nt population, the small RNAs in the IP sample could be successfully labeled at the 3′ end using RNA ligase and ^32^pCp, consistent with a free 3′-OH. As before, the small RNAs in the IP sample were resistant to labeling at the 5′ end using PNK, unless first dephosphorylated (CIP+PNK), and were able to be efficiently labeled by capping enzyme and ^32^pGTP (Figure 4C). These data indicate, that as with the bulk of the 27 nt population, the majority of the 27 nt small RNAs that associate with EhPiwi-rp have 5′-polyP termini.

**Figure 5:**
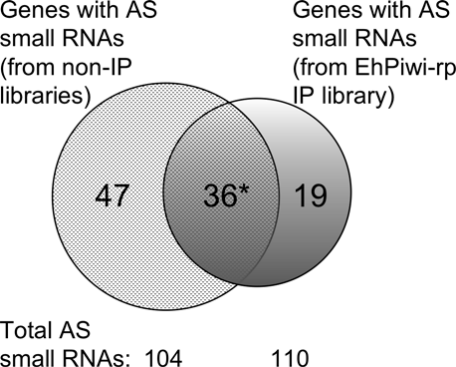
Overlap in genes targeted by antisense small RNAs in small RNA libraries made from non-immunoprecipitated and immunoprecipitated material. Of the 83 genes to which antisense small RNAs map, 36 genes were also targeted by antisense small RNAs from the Myc-EhPiwi-rp immunoprecipitated sample (p = 1.7e^−61^). * Three small RNAs cloned from the non-IP and IP libraries were highly similar (few nucleotide mismatch at the 5′ or 3′-termini of the small RNA).

In order to determine the composition of the small RNAs that IP with EhPiwi-rp, we generated a small RNA library from the EhPiwi-rp immunoprecipitated sample using a 5′P-independent method. A total of 309 small RNAs that mapped to the *E. histolytica* genome were cloned (Table S1 and Figure S2). As expected the amount of rRNA or tRNA contamination was minimal with only 6% of small RNAs mapping to these elements. A total of 36% of small RNAs mapped antisense to genes, 25% mapped to intergenic regions, 10% mapped as mixed hits, 18% mapped sense to genes, 5% mapped to repetitive regions, and 0.32% mapped to retrotransposons elements. The overall pattern, with the greatest percentage of small RNAs mapping antisense to ORFs, was the same as seen in the other *E. histolytica* small RNA libraries (Figure S2). There was also significant overlap in the genes targeted by the antisense small RNAs cloned from the non-IP and the IP libraries (Figure 5). Of the 83 genes with antisense small RNAs from the non-IP libraries, 36 genes also had antisense small RNAs cloned from the EhPiwi-rp IP library (p-value = 1.7e^−61^). Additionally, three small RNAs cloned from the IP library were highly similar (identical except for a few nucleotides at the 5′ or 3′ end) to small RNAs cloned from total RNA. Furthermore, one small RNA that was cloned from total RNA and that was previously shown to be detectable by Northern blot analysis (small RNA EHS-ID-27-1-30) (Figure 2B) was also cloned from the EhPiwi-rp IP library (EHS-IP-2-54 and EHS-IP-3-253). In *C. elegans* 5′-polyP small RNAs are in complex with an Argonaute protein, CSR-1 or with the Piwi protein, NRDE-3 for gene silencing [11],[22]. The *E. histolytica* Piwi-related protein has two conserved domains (PAZ-piwi like and Piwi), and the Piwi domain appears to contain the conserved residues necessary for slicer activity [6]. Thus, the fact that *E. histolytica* 27 nt 5′-polyP small RNAs associate with EhPiwi-rp strongly suggests that these small RNAs could be part of a silencing complex in *E. histolytica*.

### A substantial portion of 27 nt small RNAs map antisense to predicted open reading frames and are biased towards the 5′ ends of genes

In order to determine the genomic regions to which cloned small RNAs mapped, we performed BLAST analysis of the small RNA sequences to the *E. histolytica* genome sequence. No significant differences were identified in the overall characteristics of the genomic regions to which the small RNAs mapped, regardless of whether they were cloned in a 5′-phosphate dependent or 5′-phosphate independent manner (Figure S2). Of the 847 unique small RNAs that were cloned, 38% were identical to ribosomal and tRNA sequences, 25% mapped antisense to predicted open reading frames (ORFs), 16% mapped to intergenic regions, 8% matched multiple genomic loci, 9% matched the sense strand of ORFs, 4% mapped to repetitive regions, and 0.5% mapped to retrotransposon elements. Sequences from ribosomal RNAs, tRNAs, and sense tags to highly expressed loci are often found in small RNA libraries. For these RNAs we have no means to distinguish between biological effects and degradation products; thus, they were not further considered in our analysis. The paucity of matches to retrotransposons, especially in light of the large number of retrotransposons in *E. histolytica* was in contrast to other parasitic systems such as *G. intestinalis* and *T. brucei* where the majority of small RNAs appear to be derived from retrotransposons [15],[23],[24],[25].

**Figure 6:**
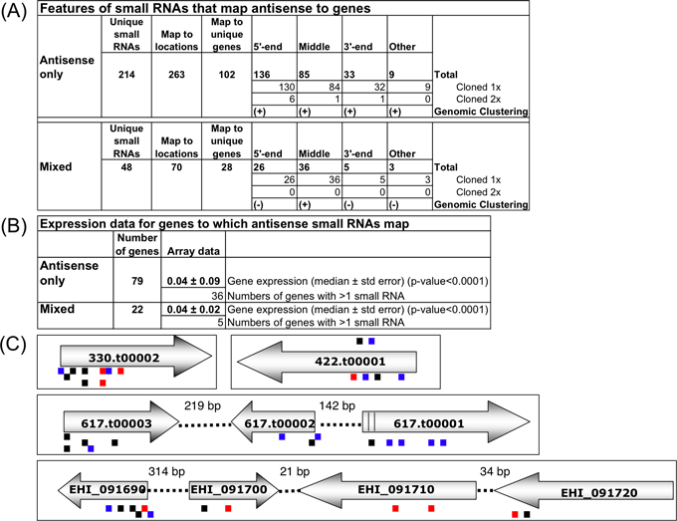
A substantial portion of *E. histolytica* small RNAs map antisense to predicted open reading frames and are associated with low expression of the cognate gene. (A) Features of small RNAs that map antisense to genes. 214 unique small RNAs map antisense to genes at 263 genomic locations, and to 102 unique genes. 48 unique small RNAs map to multiple genomic locations, including mapping antisense to genes at 70 genomic locations, and to 28 unique genes. The locations to which small RNAs map (5′ region, middle, or 3′ region) and small RNA abundance (cloned 2x) are listed. Small RNAs that map to UTRs or intron are listed under “other.” Evidence of genomic clustering is listed. (B) Features of genes to which small RNAs map antisense. The median expression level±standard error of the 79 genes to which antisense small RNAs map and the 22 genes to which antisense small RNAs that map to mixed locations are shown. The numbers of genes with >1 small RNA mapping to them are shown. (C) Schematic of small RNAs and their patterns of mapping to amebic genes. Small RNAs that map antisense to genes are shown below genes; small RNAs that map sense to genes are shown above the gene. A substantial portion of small RNAs map to the 5′ ends of genes. Multiple, non-overlapping small RNAs are identified for some genes and genomic clustering (adjacent genes with antisense small RNAs) is observed. Small RNAs cloned in a 5′-P dependent manner are shown as small black lines; small RNAs cloned in a 5′-P independent manner are shown as small red lines; small RNAs cloned from the Myc-EhPiwi-rp immunoprecipitated sample are shown as small blue lines. Genes are shown as large grey arrows and are represented by .t or EHI numbers. UTR is represented by |||. Small RNAs are drawn to scale relative to their mapped loci.

Since secondary siRNAs in *C. elegans* are largely antisense to coding regions, we focused our analysis on *E. histolytica* small RNAs that map antisense to predicted genes (Figure 6 and Table S2). Small RNAs cloned from either the 5′-phosphate dependent or 5′-phosphate independent manner and that mapped antisense to predicted ORFs were analyzed. A total of 214 unique small RNAs mapped antisense to predicted genes and a substantial portion (∼55%) mapped to the 5′ end of ORFs or very close upstream of the start codon, in the presumptive 5′-UTR (Figure 6A and 6C). Upon closer inspection, we identified that for several genes multiple, non-overlapping, antisense small RNAs were identified. The limited sequence data does not allow us to address the issue of “phasing” previously noticed for secondary siRNAs in *C. elegans*, however, the multiple non-overlapping small RNAs identified for a given gene suggests that this may also occur in *E. histolytica* [7],[8]. The small RNAs that map antisense to genes appear to be an abundant population since ∼4% of the small RNAs were cloned twice. An alternate explanation is that certain small RNAs were cloned multiple times due to biases with cloning or PCR amplification.

We identified genomic regions in which clusters of genes all had antisense small RNAs mapping to them (Figure 6A and 6C). Given the identification of genomic clustering without performing deep sequencing it would appear that this phenomenon is relatively common in *E. histolytica*. Genomic clusters of genes are coordinately regulated in response to stress and development in *E. histolytica* [21]. Whether the siRNA mechanism controls expression of adjacent genomic loci is not currently known. Of the 120 genes, which had small RNAs mapping antisense to them, 15% also had small RNAs, which mapped in the sense orientation (p = 4.8e^−19^) (Table S3). In all cases, the sense and antisense small RNAs were not complementary, however, as of yet we cannot make generalizations about this phenomenon. In *C. elegans* secondary siRNAs, both sense and antisense tags were seen for genes undergoing amplified silencing [8].

In *C. elegans*, secondary siRNAs are generated by RdRP processing of mature RNA transcripts; thus, secondary siRNAs that span exon-exon junctions have been identified [7],[8]. In our work, no antisense small RNAs were identified at exon-exon junctions, but our limited sequencing makes this negative result difficult to interpret. We did identify small RNAs that mapped to intergenic regions in a genomic locus that had antisense small RNAs to adjacent genes. Since the *E. histolytica* genome is very compact (∼9,900 predicted genes in a 24 Mb genome), one possibility is that the small RNAs that map to intergenic regions represent extension of amplified silencing that occurs downstream of a trigger, a phenomenon noted in *C. elegans* [7],[8],[15]. Alternatively, the intergenic small RNAs could map to unannotated genes.

Forty eight unique small RNAs mapped to multiple genomic regions, including antisense to predicted ORFs (Figure 6A). Small RNAs that mapped to multiple genomic loci are likely those that map to closely related gene families or repetitive regions. The overall features of these small RNAs: mapping to the 5′ ends of genes and association with low gene expression of the cognate gene (see below) were similar to the small RNAs that exclusively map antisense to genes.

The cumulative data on the *E. histolytica* 27 nt small RNAs (5′ polyphosphate termini, antisense orientation, and bias towards 5′ ends of genes) makes them sufficiently similar to secondary siRNAs involved in amplification of silencing in *C. elegans* [7],[8] so that we will subsequently refer to this fraction of small RNAs as siRNAs.

### Gene and small RNA expression are inversely correlated

In order to determine if the 27 nt 5′-polyP small RNAs that map antisense to genes have any effect on gene expression of the ORF to which they map, data from whole genome expression profiling were analyzed. A custom short oligonucleotide microarray (Affymetrix) has probes for 9,435 amebic genes and was previously used to generate expression profiles of *E. histolytica* trophozoites from three strains (HM-1:IMSS, 200:NIH, and Rahman) [21]. The data demonstrate that the genes to which antisense small RNAs map had extremely low expression values in *E. histolytica* HM-1:IMSS trophozoites, the strain and stage of the parasite from which they were cloned (Figure 6B and Table S3). The median normalized expression value for all genes on the array was 0.65±0.16 (median±standard error). The expression of genes to which antisense small RNAs mapped was 0.04±0.09 (median±standard error) (p<0.0001 compared to expression data for all genes on the array). The expression of genes that had small RNAs from the “mixed” category mapping antisense to them was 0.04±0.02 (median±standard error) (p<0.0001 compared to expression data for all genes on the array). A total of 6 genes to which antisense small RNAs map were expressed, but 4 of these were represented by probe sets that completely cross-hybridize with other genes (as indicated by a probe set identifier that ends with *_*s_at), making it difficult to address the expression of a specific gene. Considering that >80% of genes were expressed under trophozoite conditions [21], the genes to which antisense small RNAs map have significantly disproportionately low expression profiles. A subset of genes have both antisense small RNAs and sense small RNAs that mapped to them. In these instances, the location of the sense small RNAs was biased towards the middle and 3′-end of the gene, in contrast to the 5′ bias of the antisense small RNAs (data not shown). In instances of *C. elegans* amplified silencing, sense small RNAs were also identified [8]. Consistent with that data, genes that have antisense and sense small RNAs have low gene expression level (Table S3).

We analyzed the expression profiles of genes to which antisense small RNAs map from trophozoites of other strains including *E. histolytica* 200:NIH and *E. histolytica* Rahman [21]. The data demonstrate that some genes to which antisense small RNAs map are expressed in trophozoites of other *E. histolytica* strains (Table S3). Of the 101 genes with antisense small RNAs, 73 were not expressed in any of the three *E. histolytica* strains, 11 were expressed in one strain, 11 were expressed in two strains, and 6 were expressed in all strains (4 of these were represented by cross-hybridizing probes).

**Figure 7:**
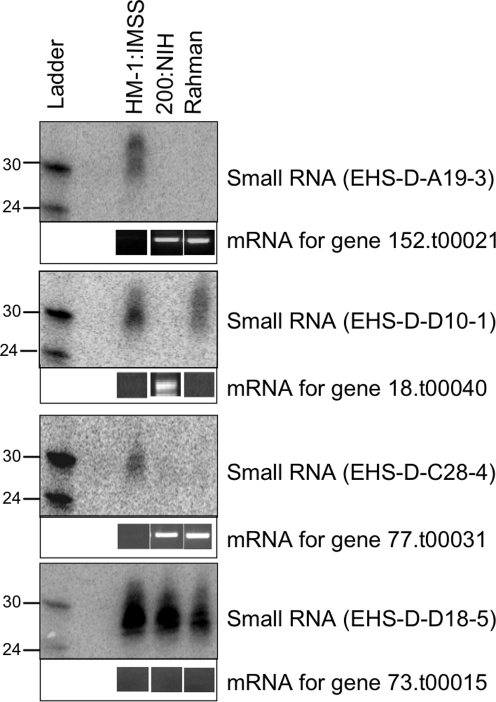
Small RNA expression levels correlate inversely with mRNA levels. A Northern blot (100 µg small RNA enriched sample each from *E. histolytica* trophozoites of strain HM-1:IMSS, 200:NIH, and Rahman) was probed with oligonucleotides to detect the expression level of the small RNA of interest. The same blot was stripped and probed for each small RNA as listed. RT-PCR for each gene from the appropriate strain is shown below the small RNA Northern blot. All controls for the RT-PCR reactions gave appropriate results.

In order to determine if there was a correlation between the presence of a small RNA and the corresponding expression level of its putative target gene (the ORF to which it maps), we performed Northern blot analysis of small RNAs where the associated gene had variable expression between amebic strains. We identified that for a given gene, small RNAs were detectable in *E. histolytica* strains with low gene expression but were not detectable in *E. histolytica* strains with high gene expression (Figure 7). The expression data for these genes were confirmed using semi-quantitative reverse transcriptase polymerase chain reaction (RT-PCR) and in all cases the results matched the array data. We noted that some small RNAs mapped at sizes larger than 30 nt. Further analysis of *E. histolytica* trophozoite small RNAs of >30 nt indicates that some ∼32 nt small RNAs have 5′-polyP structure (Figure S3 and data not shown). In selected cases, we sequenced the relevant genomic region in *E. histolytica* 200:NIH or *E. histolytica* Rahman strains and confirmed that the regions encompassing the small RNAs were identical among the three *E. histolytica* strains (data not shown). Thus, the lack of detection of a small RNA in a given amebic strain was not due to genomic sequence divergence in this strain. The inverse correlation between the *E. histolytica* 27 nt 5′-polyP small RNAs and gene expression level and the association of these small RNAs with EhPiwi-rp strongly suggests parallel functions of amebic and *C. elegans* siRNAs in mediating target silencing.

## Discussion

The work presented herein suggests that the process of gene silencing, mediated by small RNAs with 5′-polyphosphate termini, is evolutionarily conserved in the single celled parasitic eukaryote *Entamoeba histolytica*. We have identified an abundant repertoire of ∼27 nt small RNAs in *E. histolytica* with features reminiscent of secondary siRNAs in *C. elegans*, including 5′-polyP termini and antisense orientation to genes. The 27 nt small RNAs are in association with EhPiwi-related protein and an inverse correlation between small RNA and gene expression was noted suggesting that these small RNAs could mediate gene silencing. This identification of small RNAs with 5′-polyP termini in *E. histolytica* indicates that this mechanism of gene regulation is functional in single celled eukaryotes.

An increasing number of distinct small RNA species are being identified. The majority are processed by Dicer, an RNaseIII endonuclease, which generates 5′-monophosphate termini, a feature typical of siRNAs, miRNAs, tasiRNAs, tncRNA, and scnRNA [6]. However, recently small RNAs that could be generated independently of Dicer processing (rasiRNA, piRNA, and secondary siRNA) have been described [6],[7],[8]. To date, the only siRNA species proven to have 5′-polyphosphate termini are the siRNAs from *C. elegans*. These small RNAs are generated by RdRP amplification of an initial trigger siRNA [7],[8] and preferentially associate with an Argonaute protein, CSR-1, to mediate gene silencing via slicer activity [11]. Importantly, polyphosphate small RNAs are more effective than primary monophosphate small RNAs in inducing slicer activity of CSR-1 and are thus more robust at cleaving target mRNA [11].

In *E. histolytica* 27 nt RNAs with 5′-polyphosphate termini associate with EhPiwi-related protein and appear to silence genes in a strain-specific manner. We observed that genes to which these antisense small RNAs map are not expressed under trophozoite conditions, in the strain and stage of the parasite from which the small RNAs were cloned. However, a number of these genes are expressed in other parasite strains. An inverse correlation between small RNA and gene expression, and the association of the 27 nt 5′-polyP small RNAs with a Piwi-related protein, strongly suggests that the 27 nt small RNAs mediate gene silencing in *E. histolytica*. Direct proof of the roles of small RNAs in regulating amebic gene expression has been hampered due to significant issues with genetic manipulation of *E. histolytica* strains (unpublished data, H. Zhang, GM Ehrenkaufer, and U. Singh).

One difference between *E. histolytica* and *C. elegans* secondary siRNAs is their size: the *C. elegans* secondary siRNAs are ∼22 nt, whereas the *E. histolytica* counterparts are ∼27 nt. What determines the sizes of the secondary small RNAs in either species is not known; whether the size reflects an inherent feature of the RdRP or whether other endonucleases cleave secondary siRNAs needs further investigation. In *E. histolytica*, the siRNAs are a highly abundant endogenous population and were readily identified by limited sequencing. The molecular mechanisms that generate these abundant small RNAs is not known but some architectural features of the *E. histolytica* genome, including small intergenic regions and an AT rich genome with cryptic TATA-like promoter elements potentially mediating bi-directional transcription, may be contributing factors. One possibility is that in *E. histolytica* abundant secondary siRNAs are needed to deal with aberrant transcripts, which occur due to specific features of the ameba genome as outlined above.

Our preliminary analysis of the *E. histolytica* trophozoite 22 nt and 16 nt small RNA populations indicates that they likely do not have a 5′-polyP termini, however further investigations will be needed to more clearly define the features of these small RNA populations (unpublished data, H. Zhang and U. Singh). Thus far, we have not identified a small RNA species consistent with the trigger siRNAs in *C. elegans* (with a 5′-monoP) likely due to the rarity of these species and our lack of deep sequencing [7],[8]. Elucidation of the mechanism by which the 5′ polyP small RNAs are produced should shed some light on the nature and/or existence of such a trigger siRNA in *E. histolytica*. Based on functional similarity, we presume that similar trigger siRNAs exist in *E. histolytica*, however, whether other small RNAs or other structures function as triggers for the secondary siRNAs in *E. histolytica* is not currently known.

In summary, *Entamoeba histolytica*, a protozoan parasite, is the first single celled eukaryote in which gene silencing via siRNAs with 5′-polyphosphate termini has been described. Based on analogies with the amplified silencing pathway as described in *C. elegans*, our data suggest that this silencing pathway is broadly evolutionarily conserved.

## Materials and Methods

### Parasite culture, RNA extraction, and generation of transgenic parasite strains

*Entamoeba histolytica* trophozoites (HM-1:IMSS, 200:NIH, and Rahman strains), *E. dispar* SAW760 and *E. invadens* IP-1 were grown under standard conditions as previously published [21],[23],[26]. Amebae were harvested in mid log phase and small RNA extracted using a mirVana kit (Ambion). N-terminal Myc tagged constructs for EhPiwi-rp, EhRNaseIII, EhRdRP, and GFP were generated. Briefly, full-length coding regions were PCR amplified and cloned into a vector to express a N-terminal Myc tag [27]. *E. histolytica* HM-1:IMSS parasites were transfected using previously published protocols, stable transfectants selected with 12–24 µg/ml G418, and western blot analysis performed using standard protocols [23],[28]. Anti-Myc antibody (Cell Signaling) was used at 1∶1000 dilution; Anti-Lgl antibody (kind gift of William Petri) was used at 1∶50 dilution.

### Cloning and sequencing small RNAs

In order to visualize small RNAs in *E. histolytica* trophozoites, we fractionated 100 µg of total RNA with a YM100 column, resolved the sample on a 12% denaturing polyacrylamide gel, and stained the gel with SYBR gold. Small RNA cloning was based on two published protocols [8],[10]. For the 5′-P independent method, 100 µg of small RNA enriched sample was resolved on a denaturing 12% polyacrylamide gel, the 27 nt RNA fraction gel extracted, ligated to a 3′ adapter oligonucleotide (5′ rAppCTGTAGGCACCATCAAT/3ddC/ 3′) (3′-terminal dideoxy-C (ddC) base) (RT, 4 hours) and the product gel purified. The material was subjected to RT (Superscript II, 42°C, 30 min), treated with Exonuclease-I (37°C, 1 hour), a second 3′ ligation performed (5′ rAppCACTCGGGCACCAAGGA/3ddC/-3′) (RT, 4 hours), and the product gel purified. The material was PCR amplified using the adaptor primers, the final PCR products concatamerized and cloned into the pCR2.1-TOPO vector (Invitrogen) vector for sequencing. For the 5′-phosphate dependent method, 150 µg of small RNA enriched material was fractionated on a denaturing 12% polyacrylamide gel, RNA from 15–30 nt purified, and ligated to the 3′ adapter oligonucleotide (5′-rAppCTGTAGGCACCATCAAT/3ddC/-3′, 3ddc = 3′-terminal dideoxy-C base) and the 5′ adapter oligonucleotide (5′-TCGTAGGCACCTGaaa-3′; uppercase = DNA, lowercase = RNA). After reverse transcription, two rounds of PCR (32 cycles total) were performed, the PCR products concatamerized, cloned and sequenced as outlined above. The library generated from the EhPiwi-rp immunoprecipitated sample was made in a 5′-independent manner (personal communication, Sam Gu and Andy Fire). Briefly, RNA was extracted from immunoprecipitation, directly ligated to the 3′ adapter oligonucleotide, purified, treated with CIP and PNK, and ligated to the 5′ adapter oligonucleotide. Subsequent steps were as for the 5′-P dependent cloning method as outlined above.

### BLAST analysis

The small RNA sequences were extracted and BLASTed against the *E. histolytica* HM-1:IMSS genome sequence (http://www.tigr.org/tdb/e2k1/eha1/) (http://pathema.jcvi.org/cgi-bin/Entamoeba/PathemaHomePage.cgi) using search settings for short and nearly exact matches (expect = 1,000, Word size = 7). Additionally, all *E. histolytica* sequences annotated as belonging to the SINE/LINE retrotransposons were downloaded and analyzed by BLAST analysis. Sequence tags with 100% match, 1 mismatch, or up to 2 mismatches (1 terminal and 1 internal mismatch) to the *E. histolytica* genome sequence were analyzed further [29]. Antisense small RNAs are categorized as follows: map to the first 25% of a coding region (map to the 5′ of a gene); map to the last 25% of a coding region (map to the 3′ of a gene); and map to the middle 50% of a coding region (map to the middle of a gene). Small RNAs were considered to have genomic clustering if ≥ 2 adjacent genes had antisense small RNAs. The untranslated region (UTR) of a gene was defined as the 50 bp region upstream or downstream of a predicted start or stop codon respectively.

### Northern blot analysis

High resolution Northern blot analysis was done using standard protocols [30]. Briefly, 10 µg–100 µg of RNA was separated on a denaturing 12% polyacrylamide gel, transferred to a membrane, probed with end-labeled ^32^P-labeled oligonucleotides in perfectHyb buffer (Sigma) at 37°C and washed using low (2X SSC, 0.1% SDS at RT for 15 min) and medium (1X SSC, 0.1% SDS at 37°C for 15 min) stringency conditions. Standard Northern blot analysis was done using 10 µg of total RNA resolved on a denaturing 1.2 % agarose gel, probed with end-labeled ^32^P-labeled oligonucleotides in perfectHyb buffer (Sigma) at 37°C and washed using low stringency conditions according to manufacturer's instructions.

### Small RNA structure analysis and immunoprecipitation experiments

Biochemical analysis of small RNAs were performed using standard methods [7],[8],[11]. Briefly, the structure of the 3′ termini was determined with an RNA ligation reaction using T4 RNA ligase (NEB) and α-^32^P pCp (RT; 2 hours). Additionally, potential modifications at the 3′-OH termini were identified in a β-elimination reaction by treating 10 µg of small RNA with sodium periodate (25 mM, RT, 10 min), followed by heating to 45°C for 90 min. The 5′-termini were analyzed by CIP, PNK, Capping enzyme, and Terminator treatment [7],[8],[11]. Calf intestinal alkaline phosphatase (NEB) was used to treat RNA (37°C; 1 hour), followed by phenol/chloroform extraction. T4 Polynucleotide Kinase (NEB) was used to treat RNA (37°C; 1 hour). Capping was assessed with Guanylyltransferase (Ambion), the vaccinia virus capping enzyme, which was used to add α-^32^P-GTP to RNA product (37°C; 1 hour). For Terminator susceptibility, the RNA sample was treated with Terminator enzyme (30°C; 1 hour). A control sample (synthetic RNA 5′-end labeled with ^32^P and a 3′-OH terminus) was added to the RNA material, the combined sample resolved on a 12% polyacrylamide gel, and probed with a radiolabeled probe to detect the small RNA of interest.

For immunoprecipitation experiments, α-Myc antibody was incubated with parasite lysate (2 hours, 4°C) washed x2 and pelleted. RNA was isolated (mirVANA kit), labeled with PNK, ^32^P-pCp, and Capping enzyme as outlined above and resolved on a 12% denaturing polyacrylamide gel.

### *E. histolytica* microarray analysis and reverse transcriptase-polymerase chain reaction (RT-PCR) confirmation of microarray data

A custom Affymetrix DNA microarray for *E. histolytica* with probe sets representing 9,435 ORFs has previously been used to determine the expression profile of *E. histolytica* trophozoites of HM-1:IMSS, 200:NIH, and Rahman strains [21]. For RT-PCR, total RNA was extracted from log phase *E. histolytica* parasites of the appropriate strain using a mirVana reagent kit (Ambion). cDNA was made using SuperScript II Reverse Transcriptase kit (Invitrogen), including a DNAse treatment before RT. PCR was performed with serial 10-fold dilutions of cDNA and a negative RT control was included with each reaction. Four genes, 152.t00021, 18.t00040, 77.t00031, and 73.t00015 were analyzed by RT-PCR. Primers used are listed in Table S4. The ssRNA gene was used as a loading control [21].

### Statistical analyses

Statistical analyses were done using an unpaired Student's t-test (comparison of expression data for genes with antisense small RNAs compared to all genes in the genome) or a hypergeometric distribution in R downloaded from the BioConductor project (http://www.bioconductor.org) (comparison of sense small RNA distribution).

## Supporting Information

Figure S1 Small RNA populations are readily observed in trophozoites of *Entamoeba dispar* and *Entamoeba invadens*. Three endogenous small RNA populations (∼27 nt, ∼22 nt, and ∼16 nt) can be detected in *E. histolytica*, *E. dispar* and *E. invadens* trophozoites by SYBR gold staining. The 27 nt population is the most abundant.
(1.82 MB TIF)

Figure S2 Summary of genomic loci to which small RNAs map. The number of tags in each category and % distribution of total are indicated for each category. Small RNAs that map antisense to coding regions are indicated by grey shading. (A) Small RNAs cloned in a 5′-phosphate dependent manner. 342 unique small RNA sequences match to the *E. histolytica* genome sequence. (B) Small RNAs cloned in a 5′-phosphate independent manner. 196 unique small RNA sequences match to the *E. histolytica* genome sequence. (C) Small RNAs cloned in a 5′-phosphate independent manner from the Myc-EhPiwi-rp immunoprecipitated sample. 309 unique small RNA sequences match to the *E. histolytica* genome sequence. (D) Combined list of 847 unique cloned small RNAs that map to the *E. histolytica* genome sequence.
(5.82 MB TIF)

Figure S3 Sizes of small RNAs cloned in a 5′-phosphate dependent manner peak at ∼16 nt size but map at ∼27 nt when tested by Northern blot analysis. (A) The size distribution of the cloned small RNAs. The number of cloned small RNAs is indicated on the y-axis and the size of the cloned RNAs is listed on the x-axis. (B) All cloned small RNAs are detected at ∼27 nt by Northern blot analysis, regardless of the size at which they were cloned (indicated below Northern blot results). 10–100 µg of total RNA from *E. histolytica* trophozoites was probed with end-labeled 32P oligonucleotides corresponding to the cloned small RNAs. L = ladder; R = RNA.
(5.82 MB TIF)

Table S1 Sequence of all unique small RNAs identified in *E. histolytica* HM-1:IMSS trophozoites. (A) Small RNAs identified by 5′-phosphate dependent cloning. (B) Small RNAs identified by 5′-phosphate independent cloning. (C) Small RNAs identified by 5′-phosphate independent cloning from the Myc-EhPiwi-rp immunoprecipitated sample.
(0.16 MB XLS)

Table S2 Overview of small RNAs that map antisense to open reading frames. The small RNA name, sequence, size, gene that it maps to, gene accession number, and contig coordinates are shown. Location refers to the portion of the gene to which the small RNA maps: 5 = 5′ region of gene, 3 = 3′ region of gene, M = middle of gene, intron, or UTR. (A) Small RNAs identified by 5′-phosphate dependent cloning. (B) Small RNAs identified by 5′-phosphate independent cloning. (C) Small RNAs identified by 5′-phosphate independent cloning from the Myc-EhPiwi-rp immunoprecipitated sample.
(0.09 MB XLS)

Table S3 Microarray data for all genes to which antisense small RNAs map. The probe ID, locus number, gene description, and normalized microarray expression data for trophozoites from three *E. histolytica* strains (HM-1:IMSS, 200:NIH, and Rahman). Probe sets in the first column are those genes, which had antisense small RNAs map exclusively to them. Probe sets in the second column are those genes which had antisense small RNAs that mapped to them as well as to other regions of the *E. histolytica* genome. Genes with detectable expression levels are labeled in green boxes. Genes in bold are analyzed for associated small RNAs in Figure 7. Genes which have sense small RNAs also cloned are indicated. Probes with _s_at cross hybridize completely to other genes in the genome; _x_at are probes with some cross-hybridization; _at probes are unique to that gene. Obtained from previously published data [21].
(0.04 MB XLS)

Table S4 The primers (listed 5′-3′), templates for PCR and annealing temperature are listed.
(0.05 MB PDF)
