# Supplementary figures and images for: Small RNAs with 5′-Polyphosphate Termini Associate with a Piwi-Related Protein and Regulate Gene Expression in the Single-Celled Eukaryote Entamoeba histolytica
Source: PLoS Pathog. 2008 Nov 28;4(11):e1000219. doi: 10.1371/journal.ppat.1000219 (PMC2582682; doi:10.1371/journal.ppat.1000219)

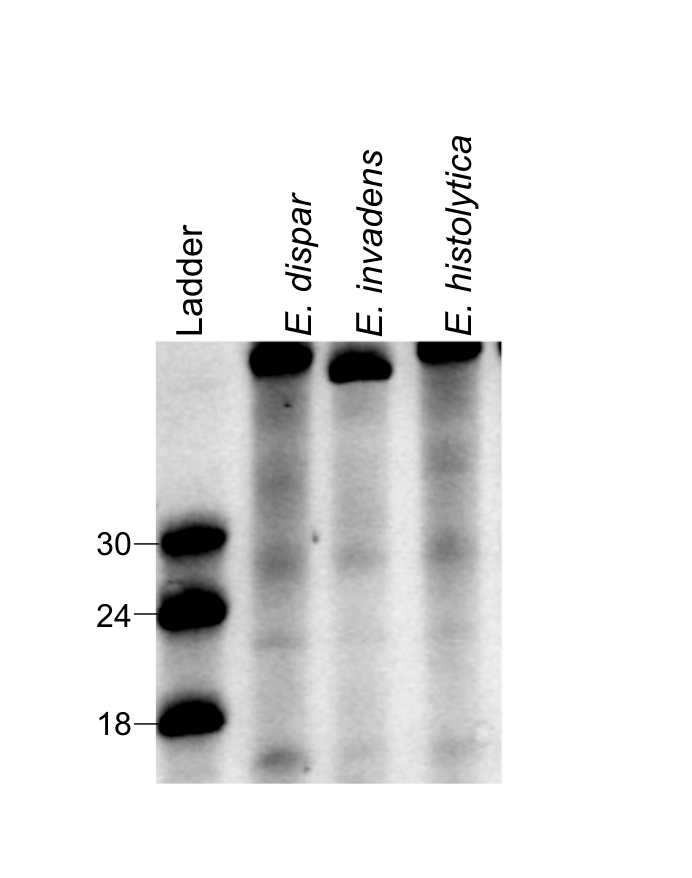

Supplement: Figure S1 — Small RNA populations are readily observed in trophozoites of Entamoeba dispar and Entamoeba invadens. Three endogenous small RNA populations (∼27 nt, ∼22 nt, and ∼16 nt) can be detected in E. histolytica, E. dispar and E. invadens trophozoites by SYBR gold staining. The 27 nt population is the most abundant. (1.82 MB TIF) [file ppat.1000219.s001.tif]

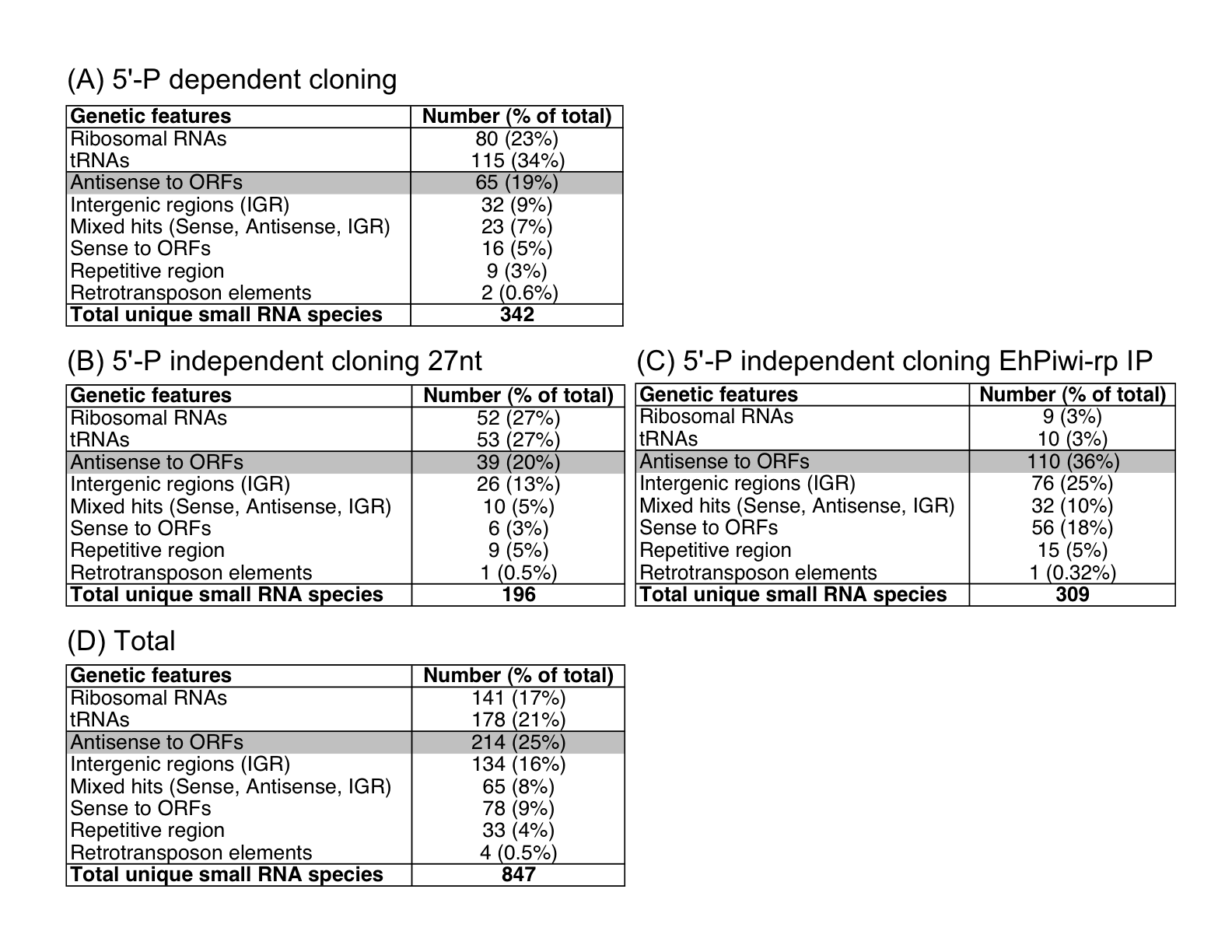

Supplement: Figure S2 — Summary of genomic loci to which small RNAs map. The number of tags in each category and % distribution of total are indicated for each category. Small RNAs that map antisense to coding regions are indicated by grey shading. (A) Small RNAs cloned in a 5′-phosphate dependent manner. 342 unique small RNA sequences match to the E. histolytica genome sequence. (B) Small RNAs cloned in a 5′-phosphate independent manner. 196 unique small RNA sequences match to the E. histolytica genome sequence. (C) Small RNAs cloned in a 5′-phosphate independent manner from the Myc-EhPiwi-rp immunoprecipitated sample. 309 unique small RNA sequences match to the E. histolytica genome sequence. (D) Combined list of 847 unique cloned small RNAs that map to the E. histolytica genome sequence. (5.82 MB TIF) [file ppat.1000219.s002.tif]

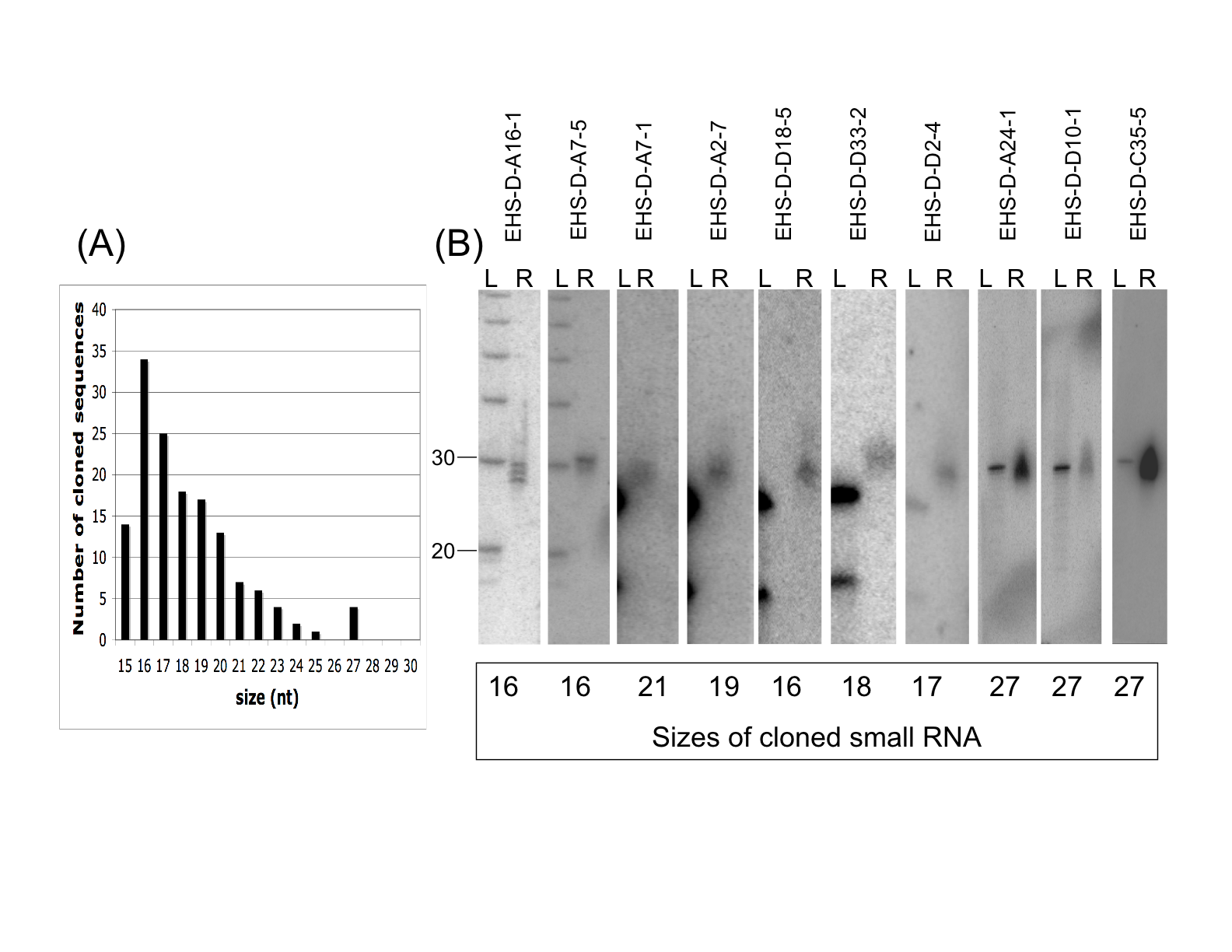

Supplement: Figure S3 — Sizes of small RNAs cloned in a 5′-phosphate dependent manner peak at ∼16 nt size but map at ∼27 nt when tested by Northern blot analysis. (A) The size distribution of the cloned small RNAs. The number of cloned small RNAs is indicated on the y-axis and the size of the cloned RNAs is listed on the x-axis. (B) All cloned small RNAs are detected at ∼27 nt by Northern blot analysis, regardless of the size at which they were cloned (indicated below Northern blot results). 10–100 µg of total RNA from E. histolytica trophozoites was probed with end-labeled 32P oligonucleotides corresponding to the cloned small RNAs. L = ladder; R = RNA. (5.82 MB TIF) [file ppat.1000219.s003.tif]
