# Supplementary material for: Small RNAs with 5′-Polyphosphate Termini Associate with a Piwi-Related Protein and Regulate Gene Expression in the Single-Celled Eukaryote Entamoeba histolytica
Source: PLoS Pathog. 2008 Nov 28;4(11):e1000219. doi: 10.1371/journal.ppat.1000219 (PMC2582682; doi:10.1371/journal.ppat.1000219)
Supplement: Table S4 — The primers (listed 5′-3′), templates for PCR and annealing temperature are listed. (0.05 MB PDF) [file ppat.1000219.s007.pdf]

Supplementary Table 4

Primers (5′--3′)	Template	Annealing Temp
EhPiwi-rp sense: CCCGGGCAACCAT CAATTCACG	gDNA	55-60° C
EhPiwi-rp antisense: CTCGAGTTAATAATAAGGATGTG		
EhRNaseIII sense: CCCGGGAGCTCAA CTACATTA	cDNA	50° C
EhRNaseIII antisense: CTCGAGTTATTGTGATGGATGAAC		
EhRdRP1 sense: CCCGGGTATAGATT CTTAATTTCT	gDNA	55° C
EhRdRP1 antisense: CTCGAGTTATTTAATTTGTTTTAGT		
GFP sense: AGATCTATGAGTAAAGGAGAAGAACTTTTCA	plasmid	55° C
GFP antisense: CTCGAGCTATTTGTATAGTTCATCCATGCCATG		
Kinase sense: CCCGGGAGTTTTATCTTTGCAAAAAAAGAGAGC	cDNA	53° C
Kinase antisense: CTCGAGTTATGAAAAATATTCTCTAAATAATTGTAC		
77.m00175-F: GGAGTTGGACCATCAGGAGA	cDNA	55° C
77.m00175-R: GCCCCGTGTTTTCCTAAAAT		
73.m00152-F: TTGCTGGTTGTTTGTTGTTTG	cDNA	55° C
73.m00152-R: GCGGCATTACAAACTTGGTT		
18.m00319-F: ACTTCCACCAGGACAAATGG	cDNA	55° C
18.m00319-R: ACCACCTTGTTGTGGGTAGC		
267.m00070-F: TTTCAAAAGGATGTGGATTCG	cDNA	55° C
267.m00070-R: GGACGATTATTGCCTTTTGC		
73.m00152-F: CCAGCTACAGGAGCTCAACC	cDNA	55° C
73.m00152-R: TTGTCCACTCATTCCAGCAG		
